# The Association of Peptide Hormones with Glycemia, Dyslipidemia, and Obesity in Lebanese Individuals

**DOI:** 10.3390/metabo12111051

**Published:** 2022-11-01

**Authors:** Murielle Abou-Samra, Koen Venema, Carole Ayoub Moubareck, Mirey Karavetian

**Affiliations:** 1 Faculty of Health, Medicine and Life Sciences, School of Nutrition and Translational Research in Metabolism (NUTRIM), Maastricht University, 6200 MD Maastricht, The Netherlands; 2 Centre for Healthy Eating & Food Innovation, Faculty of Science & Engineering, Maastricht University Campus Venlo, 5928 SZ Venlo, The Netherlands; 3 College of Natural and Health Sciences, Zayed University, Dubai 19282, United Arab Emirates; 4 Department of Food and Nutrition, Toronto Metropolitan University, Toronto, ON M5B 2K3, Canada

**Keywords:** obesity, insulin resistance, glucagon-like peptide-1, leptin, ghrelin, pancreatic peptide YY, cholecystokinin, dyslipidemia

## Abstract

Peptide-hormones, including pancreatic peptide-YY(PYY), glucagon-like peptide-1 (GLP-1), cholecystokinin (CCK), insulin, and leptin function as satiety signals, while ghrelin promotes hunger. These hormones are also involved in glucose homeostasis and body-weight regulation. The aim of this cross-sectional analysis was to examine the association of these peptide-hormones with obesity-markers, insulin-resistance, and dyslipidemia (total-cholesterol (TC), low-density-lipoprotein-cholesterol (LDL-C), high-density-lipoprotein-cholesterol (HDL-C), triglyceride (TG)). Sixteen-obese (OB) adults and 21 normal-weight (NW) age-and gender-matched counterparts were recruited. OB-participants showed significantly higher levels of leptin, insulin, Homeostatic-Model Assessment of Insulin Resistance (HOMA-IR), and TG. NW participants had significantly higher levels of ghrelin. GLP-1 was positively correlated with insulin, HOMA-IR, and obesity-markers except percent body fat. Leptin was positively correlated with all markers (except glucose and dyslipidemia). PYY was positively correlated with BMI, insulin and HOMA-IR. Ghrelin was inversely correlated with all of the markers except glucose, TC, and LDL-C. In the regression analysis model, leptin was positively associated with obesity markers and insulin resistance. Our results indicate a significant difference in peptide hormones among OB and NW Lebanese individuals. Since there is controversial evidence regarding body-weight and peptide-hormones in the literature, this study highlights a step forward towards finding ethnic based strategies to treat obesity and its consequences.

## 1. Introduction

Obesity has become an epidemic, whereby currently overweight/obesity poses more health risks and mortality than underweight/undernourishment [1]. Obesity is a complex condition that is caused by an imbalance between energy intake and energy expenditure, and is defined as a body mass index (BMI) greater than 30 kg/m^2^ [2]. Its prevalence is on an ever increasing rise; 42.7% of adults in the United States are obese [3], 30% to 70% in the Eastern Mediterranean region (EMR) [4], 35% in Saudi Arabia followed by 27.8% in United Arab Emirates [5], 27% in Lebanon and 11.7% in Italy [6]. The provision of obesity care has proved challenging in the EMR countries, in particular Lebanon. These challenges include lack of awareness of lifestyle change strategies for obesity treatment among the medical community and overdependence on bariatric surgery [7].

Appetite and energy homeostasis are regulated in humans through the interaction between the central nervous system, in particular the hypothalamus, and peripheral hormones [1,2,8,9]. The hypothalamus regulates energy homeostasis by integrating signals from peptide hormones in the periphery in response to a fluctuation in nutritional status, to modify food intake and metabolic rate [9]. These peripheral hormones are categorized into short term and long-term effectors. The short-term signals regulate energy balance through certain peptide hormones that are released from enteroendocrine cells during fasting or feeding [9]. These gut hormones are divided into appetite/orexigenic and satiety/anorexigenic hormones. Ghrelin is the only potent orexigenic hormone that is released during fasting from the stomach, initiates feeding, and regulates meal frequency [2,10]. The short term anorexigenic gut peptides, such as cholecystokinin (CCK), pancreatic peptide YY (PYY), glucagon like peptide-1 (GLP-1), and glucagon-like peptide-2 (GLP-2), induce satiety and meal termination [2]. As for the long-term satiety peripheral signals, leptin is secreted by adipose tissue, and insulin by the pancreas can cross the blood–brain barrier to reach the arcuate nucleus in the hypothalamus to control appetite. [1,2,8,11,12]. Their plasma levels are proportional to body fat; the more fat the higher their level is in circulation [1,2]. Several lines of research have shown that imbalances in gut hormones are found in those who are overweight or obese, and this is believed to have an effect on the development of insulin resistance, type 2 diabetes, and cardiovascular disease [11,12,13].

To date and to our knowledge, with the high obesity prevalence among the EMR populations, and in particular Lebanon, there are no studies evaluating the role of these peptide hormones on body weight, glycemia, and lipid profile in Lebanese Individuals. Thus, the aim of our study was to assess the association of peptide hormones with respect to obesity markers and glycemia and lipid profile among normal weight (NW) and obese (OB) Lebanese individuals residing in Lebanon.

## 2. Materials and Methods

### 2.1. Study Design

This was a cross sectional study, conducted in accordance with the Declaration of Helsinki and approved by the Institutional Review Board of Middle East Institute of Health University Hospital (MEIH-UH) Lebanon (AL-04-02/2018-IRB/MEIH).

### 2.2. Study Population

The study was conducted at the MEIH-UH between November 2019 and April 2021. Data were retrieved from a larger study focusing on gut microbiota composition as primary outcome profiling the change in the Firmicutes/Bacteriodetes ratio pre and post bariatric surgery. Thus, power sample calculations were based on the findings of Damms-Machado et al. (2015) where the mean (SD) Firmicutes/Bacteriodetes ratio changed significantly from 5.9 ± 2.1 to 10.4 ± 1.4 in 3 months post surgery [14]. To generate a similar significant effect of a sample size of 2 patients was estimated to achieve 80% power at a 5% alpha [15]. Expecting a drop out effect size in this protocol, we multiplied the number of required patients by 5. Thus, a sample size of 10 Lebanese participants per group was determined to be adequate. Eligibility criteria included stable body weight, free of antibiotic use (last 3 months), adult participants (18–60 years old) from both sexes. The eligibility criteria were assessed by using a screening questionnaire addressing ethnicity, general health, weight fluctuation, alcohol consumption, physical activity, medication, antibiotic and supplement use. Consenting participants with a Body Mass Index (BMI) of ≥35 kg/m^2^ were assigned to the obese (OB) study group; afterwards, normal weight (NW) individuals with a BMI of 18.5–24.9 kg/m^2^ matched for age and sex with their OB counterparts were assigned to the NW/ control group. A 24-h food diary was given to the subjects and was filled over three days to evaluate energy intake. Exclusion criteria were weight loss ≥5% in last 3 months, pregnancy, ethanol consumption of > ½ ounce (OZ)/day for females and 1 OZ/day for males [16], patients on medical treatment for diabetes, dyslipidemia and hypertension, and unwilling to provide written consent. OB subjects were recruited from the weight loss program of MEIH-UH by staff over a period of 2 years until sample saturation was achieved. As for the NW subjects, they were recruited from the general community in Lebanon, using direct approach and by word of mouth. All participants gave their informed consent for inclusion prior to participating.

Thirty-seven eligible subjects (10 males, 27 females) consented to join the study. Sixteen were OB (4 males, 12 females) and 21 NW counterparts (6 males, 15 females). Eligible subjects arrived to the MEIH-UH after a 12 h fast and were asked to avoid alcohol consumption, as well as any unusual strenuous exercise 24 h before the screening. On site, venous blood samples were collected by a registered nurse and a total of three vacutainers tubes that contained Ethylenediaminetetraacetic acid (EDTA) were used and one Red Top Blood Collection Tube. Five ml blood was collected in the Red Top Blood Collection Tube for the analysis of glucose, insulin, total cholesterol (TC), low density lipoprotein cholesterol (LDL-C), high density lipoprotein cholesterol (HDL-C), and triglyceride (TG) at the Department of Pathology and Laboratory Medicine at MEIH-UH. In one of the EDTA tubes 20 μL Pefabloc (Sigma Aldrich, St. Louis, MO, USA; 100 mg/mL in distilled water) was added to stabilize ghrelin and in another tube 20 μL DPP IV inhibitor (Sigma Aldrich) was added to 2 mL of blood to prevent the breakdown of PYY. These samples were centrifuged for 15 min at 12,298× g at 3 °C; the separated plasma was stored in aliquots at −20 °C to be used later on for measuring the levels of GLP-1, GLP-2, PYY, CCK, ghrelin, and leptin.

### 2.3. Anthropometric Measurements

While subjects wore light clothes and no shoes, height and weight were measured using “SECA” balance and BMI was calculated as the ratio of weight (Kg) to height (m) squared (kg/m^2^). Waist circumference (WC) (cm) was measured to the nearest 0.1 cm using a flexible, non-stretchable measuring tape at the umbilicus level midway between the lower ribs and the upper iliac crest while standing [17]. Normal WC according to the International Diabetes Federation (IDF) using Europids cut off values of <94 cm for males and <80 cm for females were used [17]. Waist to height ratio (WtHR) was calculated as the ratio of the waist circumference divided by height (both in cm). A cut-off of 0.5 was used to define abdominal obesity, whereby a WtHR < 0.5 was considered as normal [18]. Body composition was assessed using the Inbody body composition analyzer 720 (InBody Co., Ltd., Seoul, Korea) which uses a segmental multifrequency bioelectrical impedance analysis technique to analyze the percent body fat (%BF) that produces small individual error and can be used as a substitute when dual-energy X-ray absorptiometry is not available [19,20]. Cutoffs of ≥25% and ≥35% were used to define elevated %BF among men and women, respectively [21].

### 2.4. Biochemichal Measurements

#### 2.4.1. Glycemia

Concentrations of fasting plasma glucose were determined via an enzymatic colorimetric method using the Roche/Hitachi Cobas^®^ 6000 analyzer (Roche, Basel, Switzerland) at the Department of Pathology and Laboratory Medicine at MEIH-UH.

#### 2.4.2. Insulin

The Department of Pathology and Laboratory Medicine at MEIH-UH determined the serum concentration of insulin via a sandwich ELISA principle using the Elecsys insulin kit (Roche, Basel, Switzerland) by using Roche/Hitachi Cobas^®^ 6000 analyzer.

#### 2.4.3. Homeostatic Model Assessment for Insulin Resistance (HOMA-IR)

Insulin resistance was calculated using the HOMA index defined as (fasting immunoreactive insulin in microunits per milliliter (µU/mL) × fasting plasma glucose in milligrams per deciliter (mg/dL))/405 [22]. A cut off value of 2.32 was used as the value for healthy individuals [23].

#### 2.4.4. Blood Lipids (Total Cholesterol (TC), Low Density Lipoprotein Cholesterol (LDL-C), High Density Lipoprotein Cholesterol (HDL-C), and Triglyceride (TG))

Concentrations of fasting blood lipids (TC, LDL-C, HDL-C, and TG) were determined via an enzymatic colorimetric method using the Roche/Hitachi Cobas^®^ 6000 analyzer (Roche, Basel, Switzerland) at the Department of Pathology and Laboratory Medicine at MEIH-UH.

#### 2.4.5. GLP-1, GLP-2, PYY, CCK, Ghrelin, Leptin, and Leptin/Ghrelin Ratio

Concentrations of serum GLP-1, GLP-2, PYY, ghrelin, and leptin were measured by the use of ELISA kits from Diametra Millipore (Billerica, MA, USA), following the manufacturer’s instruction. The enzyme activity was measured spectrophotometrically by the Thermo Scientific™ Varioskan™ LUX multimode microplate reader (Thermofisher Scientific Inc., Waltham, MA, USA) set to 450 nm.

CCK was measured by use of ELISA kit from ABclonal Technology (Wuhan, China), following the manufacturer’s instruction. Samples were measured using the same microplate reader set to 450 nm.

For leptin/ghrelin ratio, serum leptin concentrations were converted from ng/m to pg/mL by multiplying by the value with 1000 and then serum leptin level was divided by the serum ghrelin levels.

### 2.5. Statistical Analysis

All analyses were performed using the Statistical Package for the Social Sciences software version 20 (SPSS Inc. Chicago, IL, USA). The normality of the data was tested using the Skewness and Kurtosis. Normally distributed data are expressed as mean (M) ± standard deviation (SD), and skewed data as median (Mdn) and interquartile range (IQR). Depending on the data distribution differences between the groups were assessed using independent samples *t*-test for normally distributed continuous variables or nonparametric test using Mann–Whitney U-test for skewed continuous variables and Chi-square for categorical values. Correlations between the gut peptides (GLP-1, GLP-2, PYY, ghrelin, leptin and insulin), obesity markers (WC, %BF, BMI, WtHR), glucose homeostasis indicators (insulin, HOMA-IR, glucose) to check for insulin resistance, and lipid profile (TC, LDL-C, HDL-C, TG), were determined by Pearson’s correlation analysis for normally distributed variables, and Spearman correlation for skewed variables. Binary logistic regression analyses were conducted using the forward method with categories of weight status, HOMA-IR, WC, WtHR, and %BF being the dependent variables and the gut peptides being the independent variables. The number of independent variables entered into each model was calculated as 10% of the group with the outcome and based on the results of the bivariate analyses (*p* < 0.25), and scientific literature. A value of *p* <0.05 was considered statistically significant.

## 3. Results

### 3.1. Participant Characteristics

The participant characteristics are presented in Table 1. A total of 21 NW control subjects (BMI between 18.5–24.9 kg/m^2^) and 16 OB (BMI ≥ 35kg/m^2^) were enrolled in this study. Majority of the subjects were females who represented 71.4% and 70.6% in the NW and OB respectively. Anthropometric measurements showed that WC, WtHR, and %BF were significantly higher in the OB group, as expected. As for energy intake there was no significant difference between the two groups (*p* = 0.123).

### 3.2. Biochemical and Gut Peptide Analyses

The biochemical and gut peptide analyses of the participants are shown in Table 2. Table 2a indicates the normally distributed variables and Table 2b for the skewed variables. OB participants showed significantly higher levels of leptin (*p* < 0.001), leptin/ghrelin ratio (*p* < 0.001), insulin (*p* < 0.001), HOMA-IR (*p* < 0.001), and TG (*p* = 0.017). NW participants had significantly higher levels of ghrelin (*p* = 0.002) and HDL-C (*p* = 0.001) compared to the OB. No significant differences were noted regarding PYY, GLP-1, CCK, GLP-2, fasting plasma glucose concentration, TC, and LDL-C.

### 3.3. Percent Body Fat and Gut Peptide Differences between the Sexes

Percent body fat and gut peptide differences between the sexes are shown in Table 3. %BF was higher in females in both lean (*p* = 0.01) and obese (*p* = 0.02) compared to males. Female lean and obese participants showed significantly higher levels of leptin (*p* = 0.001) compared to lean and obese males respectively. No significant differences were noted regarding the remaining gut peptides between the sexes.

### 3.4. Gut Peptides, Obesity Markers, Lipid Profile, and Glucose Homeostasis Indicators

Correlations between gut peptides, obesity markers (WC, %BF, BMI, and WtHR), glucose homeostasis indicators (insulin, HOMA-IR, glucose) and lipid profile (TC, LDL-C, HDL-C, and TG) are presented in Table 4.

A positive correlation was found between the following obesity markers and peptide hormones: BMI with insulin (r = 0.664, *p* < 0.001), leptin (r = 0.800, *p* < 0.001), GLP-1 (r = 0.365, *p* = 0.026), PYY (r = 0.333, *p* = 0.044), and leptin/ghrelin ratio (r = 0.809, *p* < 0.001); WC and WtHR with insulin (r = 0.757; *p* < 0.001), (r = 0.664, *p* < 0.001), leptin (r = 0.664; *p* < 0.001), (r = 0.753, *p* < 0.001), GLP-1 (r = 0.363; *p* = 0.027), (r = 0.334, *p* = 0.043) and leptin/ghrelin ratio (r = 0.813; *p* < 0.001), (r = 0.764, *p* < 0.001) respectively; and %BF with insulin (r = 0.734, *p* < 0.001), leptin (r = 0.874, *p* < 0.001), and leptin/ghrelin ratio (r = 0.871, *p* < 0.001).

As for markers of glucose homeostasis; leptin (r = 0.653, *p* < 0.001), GLP-1 (r = 0.382, *p* = 0.020), PYY (r = 0.333, *p* = 0.044), and leptin/ghrelin ratio (r = 0.762, *p* < 0.001) were positively correlated with insulin. Moreover, leptin (r = 0.658, *p* < 0.001), GLP-1 (r = 0.355, *p* = 0.031), PYY (r = 0.364, *p* = 0.027), and leptin/ghrelin ratio (r = 0.787, *p* < 0.001) showed a positive correlation with HOMA-IR.

Ghrelin was negatively correlated with BMI (r = −0.521, *p* = 0.001), WC (r = −0.575; *p* < 0.001), WtHR (r = −0.530, *p* = 0.001), %BF (r = −0.362, *p* = 0.028), insulin (r = −0.554, *p* < 0.001), and HOMA-IR (r = −0.582, *p* < 0.001).

None of the gut peptides were correlated with TC and LDL-C, and there was no significant correlation between CCK, obesity markers, glucose homeostasis indicators and lipid markers. There was a negative correlation between HDL-C, insulin (r = −0.475, *p* = 0.003) GLP-1 (r = −0.365, *p* = 0.026), and leptin/ghrelin ratio (r = −0.460, *p* = 0.004); and a positive association for ghrelin (r = 0.537, *p* = 0.001). As for TG positive correlations were seen with insulin (r = 0.363, *p* = 0.027) and GLP-2 (r = 0.404, *p* = 0.013); and negative correlation with ghrelin (r = −0.346, *p* = 0.036).

In the binary logistic regression analysis, reported in Table 5, leptin was positively associated with HOMA-IR (odds ratio (OR) = 1.056 and 95% confidence interval (CI) = 1.006–1.108), weight status (as being obese; OR = 1.219, and 95% CI = 1.013–1.467), WC (OR =1.151, and 95% CI = 1.022–1.297), WtHR (OR =1.139, and 95% CI = 1.038–1.251), and %BF (OR = 1.202, and 95% CI = 1.034–1.398). No significant associations were noted with ghrelin, and %BF, and weight status. On the other hand, ghrelin was negatively associated with HOMA-IR (OR = 0.991, and 95% CI = 0.984–0.998) and WC (OR = 0.987, and 95% CI = 0.976–0.998). No significant associations were noted with the blood lipid markers and the gut peptides and therefore these were not included in the binary logistic regression analysis.

## 4. Discussion

Obesity is one of the main risk factors for the development of type 2 diabetes, cardiovascular disease, and other non-communicable diseases, that are the leading cause of death worldwide [24]. Several lines of research have shown a link between an imbalance in gut peptide in overweight or obese individuals that may explain their underlying condition [2]. To our knowledge, the relationship between gut peptide hormones and obesity related health conditions have received little attention in the scientific literature when it comes to the Lebanese population, thus demonstrating the need for this research. Lebanon is part of the Eastern Mediterranean region and it is of surprise to find that Lebanon has a high percentage of obesity compared to other Eastern Mediterranean countries [6].

In our study, we noted a statistically significant difference in leptin, and ghrelin between OB and NW individuals, but no significant difference with respect to fasting GLP-1, GLP-2, PYY, and CCK. Our results confirm the dysregulation for some peptide hormones (leptin and ghrelin) and point out further controversy for other hormones with respect to obesity, insulin resistance and dyslipidemia [2]. A similar study to ours was conducted among lean and obese individuals in the United Arab Emirates (UAE) utilizing the same peptide hormones along with obesity markers and glycemia [25]. According to the latter study, conducted in UAE, the authors found a statistically significant difference in GLP-1, GLP-2, insulin, leptin, ghrelin, and CCK between obese and lean participants [25] and as a result it further confirmed the need for population-based research due to the difference in results.

Ghrelin is the gut peptide, which stimulates appetite, lipogenesis, and insulin resistance [1]. Plasma ghrelin levels peak during fasting and prior to a meal [26], and this induces eating. In accordance to the literature, [26,27,28] our study also showed fasting plasma ghrelin levels to be lower in OB than in NW individuals. In addition to this, ghrelin was negatively correlated with obesity markers (WC, %BF, BMI, and WtHR). These findings suggest that ghrelin levels fluctuate in accordance to a change in body weight [29]. Fasting ghrelin levels decrease with obesity and increase with weight loss [29]. This decrease leads to a physiological adaptation to the positive energy balance, rather than a causal effect [29]. Furthermore, obese individuals lack a decline in ghrelin after food intake, and as a result appetite will not be reduced and further weight gain will be observed [28,30]. In addition, we also found a negative correlation between ghrelin, insulin, HOMA-IR, and TG and a positive association with HDL-C. This negative correlation between fasting ghrelin and insulin has also been reported in previous studies [28,31,32], and our data are in line with these results and this negative correlation could eventually lead to type 2 diabetes and cardiovascular disease in obese individuals [31]. With respect to TG and ghrelin, OB individuals were found to have higher fasting TG and lower ghrelin compared to the NW. In agreement with our results, a study that was conducted on rodents, showed the inverse relationship between triglyceride and ghrelin [33]. High triglyceride levels were found to have opposite effects on leptin and ghrelin transport across the blood–brain barrier. Furthermore, triglycerides were found to decrease the transport of leptin while increasing the uptake of ghrelin into the brain and as a result this stimulated appetite and feeding [33].

In addition to our findings, hypertriglyceridemia is a risk factor for cardiovascular disease and is associated with obesity and insulin resistance [34,35]. Furthermore, when there is an increase in TG there is a reduction in HDL-C [34]. This was seen in our study where fasting TG and insulin were higher and HDL was lower in the OB compared to the NW individuals. With respect to the peptide hormones there was a positive correlation with GLP-2 and TG. It is known that GLP-1 and GLP-2 are co-secreted in response to the ingestion of the same nutrients [35]. Several studies regarding post-prandial GLP-1 and GLP-2 secretion and the production of triglyceride rich lipoprotein (TRL) have been conducted in which GLP-1 and GLP-2 have opposite effects. GLP-1 was found to decrease TRL in plasma and GLP-2 was found to do the opposite [34,35]. Based on these results and to further understand the underlying mechanism, it is important to do additional research involving GLP-2 and its effect on TG during fasting and in the postprandial state in OB and NW individuals.

As for the link between obesity and GLP-1, there is mixed evidence, with some studies showing that those who are obese have lower circulating levels of this hormone than those of normal weight, while others have not found this [25,36]. In our study, fasting GLP-1 was not found to be statistically different between the two groups. BMI, WC, and WtHR were significant predictors of fasting GLP-1 concentrations in OB subjects. Furthermore, fasting GLP-1 concentrations were positively associated with HOMA-IR and insulin. These observations may be essential features of obesity and require further investigation to elucidate the mechanisms. One can envisage higher fasting GLP-1 may increase circulating insulin, which may lead to insulin resistance among obese patients [37]. Increased adiposity as a result of high insulin has been well documented through several known mechanisms, including reduced lipolysis, increased lipogenesis and adipogenesis [38]. On the other hand, GLP-1 is secreted in response to food intake and it has a known role in the regulation of glucose homeostasis, increasing satiety, reducing gastric emptying, inducing weight loss, and as a result, improving insulin sensitivity [39]. However, GLP-1 secretion seems to be affected by the meal composition, type of nutrient and sequence consumed, and the preceding meal [39]. Therefore, it is important to also evaluate GLP-1 postprandially and its association with HOMA-IR.

Regarding PYY, it is considered to be an appetite suppressing hormone and plays a role in energy homeostasis. However, the evidence in the literature with respect to the involvement of PYY in the development of obesity is controversial and unclear. In our study there was no significant difference between the groups. On the other hand, we also found a positive correlation between PYY, BMI, and HOMA-IR. Studies conducted on humans showed that PYY concentrations were lower in obese compared to normal weight individuals, suggesting that this appetite suppressing gut peptide is associated with the development of obesity [40,41,42]. However, other human based studies have failed to reproduce this inverse relationship between PYY and adiposity [43,44,45]. It has also been shown that there is a different response in the secretion of PYY in the fasting versus the fed state in obese individuals compared to lean subjects with the obese having a lower secretion following a meal [46]. In addition to this, PYY concentrations are affected postprandially with the type of macronutrient consumed with protein having the highest PYY response followed by fat and the least by carbohydrate [46]. The mechanisms by which obese individuals have an altered PYY profile remain unknown and it might be related to a change in the synthesis, release or removal of PYY [46]. Therefore, further studies that monitor the fasting and postprandial response of PYY to different diets should be performed.

Leptin on the other hand is not a gut hormone but rather an adiposity hormone produced by adipose tissue [8]. Leptin secretion is directly proportional to lipid content in adipose tissue [47]. This was also seen in our current study with higher concentrations of fasting leptin in obese individuals compared to NW counterparts. Furthermore, as observed and well-known before, %BF between males and females differed in both groups (Table 3). Furthermore, leptin was significantly different between sexes in both groups, with higher concentrations in females than in males, as has also been observed before [48]. These two observations are related, as one of the mechanisms of higher leptin in females has been proposed to be a higher proportion of adipose tissue and increased production rate of leptin per unit mass of adipose tissue in women than in men [48]. However, since leptin is an anorexigenic hormone that promotes satiety, there are numerous studies that support the fact that obese individuals exhibit leptin resistance at the level of the brain thus disrupting the satiety response [11,25,47]. Further to this point, excessive weight loss might resolve leptin resistance [12]. In our study, in addition to the result that leptin was found to positively correlate with all obesity markers, OB subjects had higher insulin and HOMA-IR values compared to NW subjects, and a positive correlation was seen between leptin, insulin and HOMA-IR. Studies have suggested that insulin resistance can cause leptin resistance and therefore lead to obesity, and its comorbidities [11,12,49]. In addition to this, obesity leads to high levels of leptin, which acts as a cause for the release of pro-inflammatory cytokines and an exaggerated release of insulin leading to insulin resistance [49]. These findings shed light to understanding the pathophysiology of leptin resistance in humans.

Due to the inconsistency among different results of gut peptides related to leptin and ghrelin, new parameters have been evaluated in which one of them is leptin/ghrelin ratio [50]. This ratio appears to be associated with regulating hunger, in the sense that a lower ratio is associated with reducing hunger [50]. Our results are in accordance with other studies in which obese subjects had a higher leptin/ghrelin ratio in the fasting state compared to the lean counterparts [50,51]. This was evident among all of the obesity markers (BMI, WC, WtHR, and %BF). In addition to this, diabetics were found to have a higher ratio of leptin/ghrelin [51]; in our study we noticed a positive correlation with the HOMA-IR. Since both leptin and ghrelin are involved in glucose metabolism, diabetics tend to have lower ghrelin levels and higher serum leptin [52] and as a result the leptin/ghrelin ratio would be higher in diabetics. However, further studies are required on a bigger sample size with the consideration of the effect of postprandial meal on the leptin/ghrelin ratio and also comparing diabetics to nondiabetics (obese) individuals to further understand the relationship.

Strengths of the present study include focusing on Lebanese individuals that were residing in Lebanon, while the study design involved using validated obesity and glucose homeostasis markers in order to explore the association between gut peptides and obesity and insulin resistance. However, this study had limitations, one of them being a small number of participants and this might alter our results, therefore prospective studies with a large sample size should be carried out to validate our primary findings. Furthermore, since our analysis only focused on fasting peptide hormone concentrations, looking at postprandial concentrations can provide additional insight to the relationship with obesity and impaired glucose homeostasis and dyslipidemia. Furthermore, further analysis and prospective studies involving all parameters of the metabolic syndrome and the involvement of the gut peptides should be taken into consideration to identify if these differences are a consequence or cause of obesity.

## 5. Conclusions

To our knowledge this is the first study conducted in the EMR, on fasting levels of peptide hormones in OB and NW Lebanese individuals. In this study we observed significant difference in the fasting plasma peptide hormones among the obese and normal weight Lebanese individuals with a notable relationship between leptin, glucose, and obesity markers. Since there are controversial results in the literature concerning peptide hormones among obese individuals, our results further highlight a need for finding strategies that alter peptide hormones in the fasting and postprandial state in the treatment of obesity and its consequences that might be related to different ethnic backgrounds. More long-term research is required while taking into consideration the ethnic groups and the emerging role of gut peptides in obesity therapy.

## Figures and Tables

**Table 1:** Participant characteristics.

	Normal Weight	Obese	*p*
**N**	21	16	
Age (M ± SD)	39.10 ± 9.86	35.66 ± 10.37	0.31
Female (n (%))	15 (71.4)	12 (70.6)	0.81
**Anthropometric data**			
BMI (kg/m^2^) (M ± SD)	22.07 ± 1.81	38.79 ± 3.72	<0.001 *
WC (cm) (M ± SD) ^†^	82.14 ± 9.39	117.34 ± 7.88	<0.001 *
WtHR (M ± SD) ^†^	0.37 ± 0.04	0.64 ± 0.06	<0.001 *
Percent Body Fat (%) (M ± SD)	24.38 ± 6.18	45.75 ± 5.88	<0.001 *
Energy Intake (Kcal) (M ± SD)	1641.95 ± 522.96	2042.67 ± 884.40	0.123

* The statistical significance was evaluated by *t* test for independent samples for continuous variables and by Pearson’s chi-squared test for categorical variables. Bold values denote statistical significance (*p* < 0.05); † Clinical characteristics of study participants. Normal Waist Circumference according to IDF using Europids cut off values of <94 cm for males and <80 cm for females; Waist to Height Ratio normal refers to <0.5. Abbreviations: BMI: Body mass index; WC: waist circumference; WtHR: waist to height ratio, M ± SD: Mean ± Standard Deviation.

**Table 2:** (**a**) Biochemical and gut peptide analyses in normal weight vs. obese participants (mean ± SD for normally distributed data). (**b**) Biochemical and gut peptide analyses in normal weight vs. obese participants (median (IQR) for non-normally distributed data).

(a)			
Variable	NW (M ± SD) n = 21	OB (M ± SD) n = 16	*p*
Leptin (ng/mL)	14.28 ± 8.64	64.03 ± 32.79	<0.001 *
Ghrelin (pg/mL)	449.40 ± 203.11	246.82 ± 150.85	0.002 *
PYY (pg/mL)	60.42 ± 31.48	74.22 ± 47.51	0.296
GLP-1 (pM)	24.95 ± 9.59	30.98 ± 11.84	0.096
GLP-2 (ng/mL)	3.41 ± 1.37	3.89 ± 1.21	0.273
HDL-C (mg/dL)	61.38 ± 16.61	44.69 ± 9.75	0.001 *
TG (mg/dL)	105.43 ± 50.38	149.81 ± 57.65	0.017 *
**(b)**			
**Variable**	**NW (Median (IQR)) n = 21**	**OB (Median (IQR)) n = 16**	***p***
Leptin/Ghrelin ratio	24.26 (10.79–142.76)	320.10 (81.41–908.36)	<0.001 *
CCK (pg/mL)	36.54 (21.20–152.45)	38.05 (20.40–66.68)	0.927
Glucose (mg/dL)	97.00 (80–116)	98.50 (93–119)	0.133
Insulin (µU/mL)	7.77 (4.46–13.35)	20.15 (7.74–44.15)	<0.001 *
HOMA-IR	1.85 (1.05–3.82)	4.78 (1.97–11.77)	<0.001 *
TC (mg/dL)	182.00 (131–364)	179.00 (133–358)	0.902
LDL-C (mg/dL)	101.00 (50–299)	109.00 (70–290)	0.462

(a) * Bold values denote statistical significance *(p* < 0.05) between the 2 groups. All normally distributed numerical variables were calculated using Independent-Samples *t*-test, Abbreviations: PYY: pancreatic peptide YY, GLP-1: glucagon like peptide-1; GLP-2: glucagon-like peptide-2; HDL: high density lipoprotein cholesterol; TG: triglyceride; OB: obese; NW: normal weight; M ± SD: Mean ± Standard Deviation. (b) * Bold values denote statistical significance (*p* < 0.05) between the 2 groups. All skewed numerical variables were calculated using nonparametric test using Mann–Whitney U-test, Abbreviations: CCK: Cholecystokinin; HOMA-IR: Homeostatic Model Assessment for Insulin Resistance; TC: total cholesterol; LDL-C: low density lipoprotein cholesterol; OB: obese; NW: normal weight; IQR: Interquartile range.

**Table 3:** Effect of Sex on Percent body fat and gut peptide.

Variable	Females Lean (M ± SD) n = 15	Males Lean (M ± SD) n = 6	*p*	Females Obese (M ± SD) n = 12	Males Obese (M ± SD) n = 4	*p*
%BF	26.44 ± 6.09	19.22 ± 2.07	0.001 *	48.10 ± 3.51	38.70 ± 6.28	0.002 *
Leptin (ng/mL)	17.21 ± 8.43	6.98 ± 3.10	0.001 *	77.55 ± 25.05	23.47 ± 12.22	0.001 *

* Bold values denote statistical significance (*p* < 0.05) between the 2 groups. Independent-Samples *t*-test was used. Abbreviations: %BF: percent body fat; M ± SD: Mean ± Standard Deviation.

**Table 4:** Correlations between gut peptides, obesity markers, lipid profile, and glucose homeostasis indicators.

		Insulin	Leptin	GLP1	GLP2	PYY	CCK	Ghrelin	Leptin/Ghrelin Ratio
BMI	r	**0.664 ^b^**	**0.800 ^a^**	**0.365 ^a^**	0.229 ^a^	**0.333 ^a^**	0.111 ^b^	**−0.521 ^a^**	**0.809 ^b^**
	*p*	<0.001 **	<0.001 **	0.026 *	0.172	0.044 *	0.514	0.001 **	<0.001 **
WC	r	**0.757 ^b^**	**0.664 ^a^**	**0.363 ^a^**	0.296 ^a^	0.279 ^a^	−0.117 ^b^	**−0.575 ^a^**	**0.813 ^b^**
	*p*	<0.001 **	<0.001 **	0.027 *	0.075	0.094	0.490	<0.001 **	<0.001 **
WtHR	r	**0.664 ^b^**	**0.753 ^a^**	**0.334 ^a^**	0.219 ^a^	0.306 ^a^	0.042 ^b^	**−0.530 ^a^**	**0.764 ^b^**
	*p*	<0.001 **	<0.001 **	0.043 *	0.192	0.065	0.806	0.001 **	<0.001 **
%BF	r	**0.734 ^b^**	**0.874 ^a^**	0.277 ^a^	0.199 ^a^	0.303 ^a^	0.043 ^b^	**−0.362 ^a^**	**0.871 ^b^**
	*p*	<0.001 **	<0.001 **	0.097	0.238	0.068	0.802	0.028 *	<0.001 **
Glucose	r	**0.334 ^b^**	0.217 ^a^	0.142 ^a^	−0.013 ^a^	0.281 ^a^	−0.083 ^b^	**−0.423 ^a^**	**0.402 ^b^**
	*p*	0.043 *	0.196	0.400	0.940	0.092	0.624	0.009 **	0.014 *
Insulin	r	−	**0.653 ^b^**	**0.382 ^b^**	0.269 ^b^	**0.333 ^b^**	−0.092 ^b^	**−0.554 ^b^**	**0.762 ^b^**
	*p*	−	<0.001 **	0.020 *	0.108	0.044 *	0.589	<0.001 **	<0.001 **
HOMA-IR	r	**0.991 ^b^**	**0.658 ^b^**	**0.355 ^b^**	0.232 ^b^	**0.364 ^b^**	−0.104 ^b^	**−0.582 ^b^**	**0.787 ^b^**
	*p*	<0.001 **	<0.001 **	0.031 *	0.166	0.027 *	0.542	<0.001 **	<0.001 **
TC	r	0.107 ^b^	−0.039 ^b^	0.086 ^b^	0.212 ^b^	−0.122 ^b^	−0.128 ^b^	0.044 ^b^	−0.707 ^b^
	*p*	0.529	0.820	0.611	0.208	0.471	0.452	0.794	0.679
LDL-C	r	0.163 ^b^	−0.044 ^b^	0.112 ^b^	0.217 ^b^	−0.148 ^b^	−0.038 ^b^	−0.011 ^b^	−0.034 ^b^
	*p*	0.334	0.797	0.509	0.197	0.382	0.821	0.949	0.843
HDL-C	r	**−0.475 ^b^**	−0.294 ^a^	**−0.365 ^a^**	−0.293 ^a^	−0.186 ^a^	−0.085 ^b^	**0.537 ^a^**	**−0.460 ^b^**
	*p*	0.003 **	0.078	0.026 *	0.079	0.271	0.618	0.001 **	0.004 **
TG	r	**0.363 ^b^**	0.266 ^a^	0.315 ^a^	**0.404 ^a^**	0.234 ^a^	−0.127 ^b^	**−0.346 ^a^**	0.310 ^b^
	*p*	0.027 *	0.111	0.058	0.013 *	0.163	0.452	0.036 *	0.062

Spearman correlation was conducted for correlations involving insulin, CCK, HOMA-IR, total cholesterol, leptin/ghrelin ratio, and LDL-C; while Pearson correlation was conducted for correlations involving other variables: ^a^ Pearson correlation, ^b^ Spearman correlation. Abbreviations: WC: waist circumference, %BF: percent body fat; BMI: Body mass index; WtHR: waist to height ratio; HOMA-IR: Homeostatic Model Assessment for Insulin Resistance; LDL-C: low density lipoprotein cholesterol; HDL-C: high density lipoprotein cholesterol; TG: triglyceride; GLP-1: glucagon like peptide-1; GLP-2: glucagon-like peptide-2; CCK: cholecystokinin; PYY: pancreatic peptide YY. r = correlation coefficient * Bold values denote correlation is significant at the 0.05 level (2-tailed). ** Bold values denote correlation is significant at the 0.01 level (2-tailed).

**Table 5:** Binary logistic regression of predictors of markers of obesity and glucose homeostasis.

Logistic Regression	Predictors	OR	*p*	R^2^	95% CI
HOMA-IR ^a^	Leptin Ghrelin	1.056 0.991	0.027 0.007	0.652	1.006–1.108 0.984–0.998
Weight status ^a^	Leptin	1.219	0.036	0.882	1.013–1.467
WC ^a^	Leptin Ghrelin	1.151 0.987	0.021 0.020	0.818	1.022–1.297 0.976–0.998
WtHR ^b^	Leptin	1.139	0.006	0.759	1.038–1.251
%BF ^a^	Leptin	1.202	0.017	0.816	1.034–1.398

^a^ for HOMA-IR, weight status, WC, %BF: predictors entered in the model: leptin, ghrelin. ^b^ for, WtHR predictors entered in the model: leptin. Abbreviations: HOMA-IR: Homeostatic Model Assessment for Insulin Resistance; WC: waist circumference, WtHR: waist to height ratio; %BF: percent body fat. Reference category: HOMA-IR < 2.32 vs. HOMA-IR ≥ 2; Weight status: lean vs. obese Waist Circumference: appropriate WC vs. increased WC; Waist to Height Ratio: normal WtHR vs. high WtHR.

## Data Availability

The raw data presented in this study are openly available in Mendeley Data repository: https://data.mendeley.com/datasets/yd5d2gpv4c/3 (accessed on 21 October 2022) at https://doi.org/10.17632/yd5d2gpv4c.3.
